# Characterization of Autoantigens Targeted by Anti-Citrullinated Protein Antibodies *In Vivo*: Prominent Role for Epitopes Derived from Histone 4 Proteins

**DOI:** 10.1371/journal.pone.0165501

**Published:** 2016-10-27

**Authors:** Xiaobo Meng, Peyman Ezzati, Irene Smolik, Charles N. Bernstein, Carol Ann Hitchon, Hani S. El-Gabalawy

**Affiliations:** 1 Arthritis Centre, Department of Internal Medicine, University of Manitoba, RR149, 800 Sherbrook St. Winnipeg, MB, R3A 1M4, Canada; 2 Manitoba Centre for Proteomics and Systems Biology, University of Manitoba, 799 JBRC, 715 McDermot Ave, Winnipeg, MB, R3E 3P4, Canada; 3 Department of Internal Medicine, University of Manitoba, Winnipeg, Manitoba, Canada; Duke University School of Medicine, UNITED STATES

## Abstract

Anti-citrullinated protein antibodies (ACPA) have become an integral part of the clinical definition of rheumatoid arthritis, and are hypothesized to be important in the immunopathogenesis of this autoimmune disease. Several citrullinated proteins have been demonstrated to serve as candidate autoantigens for the ACPA, based on *in vitro* immune reactions between citrullinated peptides/proteins and RA sera. Yet it remains unclear whether the autoantigens identified *in vitro* are indeed directly and specifically targeted by the ACPA *in vivo*. Moreover, it is unclear whether ACPA present in RA sera are directed towards the same spectrum of autoantigens as the ACPA present within the synovial compartment. In this study, we isolated ACPA immune complexes from RA synovial fluids (SF) and sera by using immobilized cyclic citrullinated peptides (CCP3) based immune affinity, and characterized the proteins that are directly and specifically associated with them by mass spectrometry. The results demonstrate that four histone proteins are prominent ACPA autoantigens, with the frequency of detection being histone H4 (89%), H2B (63%), H3 (63%), and H2A (58%) in ACPA positive RA SF. We further demonstrate that a histone 4 peptide containing citrulline at position Cit39 was recognized by 100% of ACPA positive RA SF. An adjacent citrulline residue at Cit40 was recognized by 34% of ACPA positive RA SF. An H4 peptide containing Cit39-40 was recognized in the serum of 94% ACPA positive RA, 77% ACPA positive first-degree relatives (FDR) of RA patients, and 2.5% of healthy controls. The Cit39-40 peptide substantially blocked the ACPA reactivity in both SF and serum. Although the spectrum of ACPA we identified was limited to those isolated using immobilized CCP3 peptides, the findings indicate that H4 is a widely recognized RA autoantigen in both the synovial and serum compartments. The identification of this immunodominant ACPA epitope may be valuable in designing approaches to immune tolerance induction in RA.

## Introduction

Rheumatoid arthritis (RA) is a chronic inflammatory joint disease in which autoimmunity is felt to play an important role in its initiation and pathogenesis [1, 2]. Seminal studies performed almost 20 years ago demonstrated that autoantibodies directed towards citrullinated proteins and peptides are detectable in the majority of RA patients, although there appeared to be considerable heterogeneity in the antigens recognized [3]. These autoantibodies, now referred to as ACPA, have been shown to have a high degree of specificity and sensitivity in multiple patient cohorts worldwide, and are valuable biomarkers for RA diagnosis and prognosis.

Citrullination results from deamination, a post-translational modification catalyzed by peptidylarginine deiminases (PAD), which convert the amino acid arginine into citrulline, a *non*-essential amino acid that potentially alters protein structure and generates new epitopes that could serve as autoantigens in individuals with an appropriate genetic background [4, 5]. The first reported citrullinated protein recognized by ACPA in RA sera was filaggrin, after which the ACPA were further demonstrated to respond to citrullinated collagen type II, fibrinogen, α-enolase, vimentin, and histones [3, 6–12].

At the time of the initial identification of citrullinated filaggrin as a candidate autoantigen in RA, citrulline containing filaggrin peptides were used as a tool to test autoantibody levels in RA, despite the fact that this protein is abundant in skin, but virtually undetectable in the synovial compartment. A cyclic form of citrullinated peptide (CCP) was then developed as a substitute antigen, and proved to be a convenient approach to replace full proteins for detecting ACPA. To optimize the CCP based ACPA assay, a second (CCP2) and third (CCP3) generation of proprietary CCP were designed and mass produced by the bio-industry. These substantially increased the sensitivity and specificity of the assay for RA diagnosis. The CCP based assays are now considered the gold standard for detecting ACPA, and have been incorporated into 2010 ACR/EULAR RA classification criteria [13, 14].

Despite the wide availability of commercial assays that are designed to capture the full scope of autoantibodies and maximize sensitivity for disease detection, it remains unclear how many and what endogenous autoantigens serve to initiate and sustain this autoimmune response. Using detailed *in vitro* analyses with peptides derived from vimentin, α-enolase, fibrinogen, and other proteins, ACPA derived from patient sera display varying degrees of cross reactivity, ranging from mono-reactive to multiple reactivity with a spectrum of citrullinated peptides/proteins [15–17]. Because of these considerations, defining endogenous autoantigens *in vivo* requires an approach that directly identifies the autoantigens that are bound to the ACPA.

In order to get a better understanding of the endogenous autoantigens targeted by ACPA *in vivo*, we isolated ACPA from RA synovial fluids and analyzed the spectrum of citrullinated antigens directly and specifically bound to these autoantibodies. Our data demonstrate that histone proteins are the most widely recognized by ACPA in RA synovial fluid, and a peptide derived from the histone H4 protein is widely recognized by ACPA in the serum of RA patients and their ACPA positive FDR.

## Materials and Methods

### Patient cohorts and samples

Synovial fluids (n = 58) were collected at the time of routine clinical arthrocentesis from seropositive RA patients (n = 34), and from patients with psoriatic arthritis, reactive arthritis, undifferentiated polyarthritis, and osteoarthritis (n = 24). Samples were processed and stored at -20°C. Serum samples (n = 405) were obtained from a cohort study of Indigenous North American (INA) RA patients (n = 130), their unaffected first-degree relatives (FDR, n = 79), and Indigenous healthy controls (n = 54). Serum was also collected from Caucasian RA patients (n = 116) and Caucasian healthy controls (n = 26) (S1 Table) [18, 19]. Whole blood was collected in serum separator containers, and allowed to clot at room temperature for 30 minutes. It was then centrifuged at 2,000 x g for 15 minutes. The resulting supernatant was collected and stored at –20°C. The clinical characteristics of subjects are similar to that previously described and presented in S2 Table [19–21].

Study subjects provided written informed consent using consent documents that were approved annually by the University of Manitoba Biomedical Research Ethics Board (BREB). Also, written research agreements to undertake the study were in place with the communities of Norway House and St Theresa Point, Manitoba, Canada. In cases where a study subject preferred to use a traditional language such as Cree, Ojibway, or Ojicree, a translator assisted in obtaining the informed written consent. Stored synovial fluid samples obtained in the context of routine arthrocentesis from non-Indigenous patients with non-RA diagnoses e.g. osteoarthritis, were used based on verbal consent that was documented during the clinical encounter. The samples were subsequently used in an anonymized manner with only a diagnosis identified. The BREB in place at that time had broadly approved this approach in routine clinical practice.

### Experimental Materials

Quanta lite CCP3 IgG ELISA kits (cat# 704535) were purchased from Inova Diagnostics (San Diego, CA), Takara peptide coating kits (MK100) from Clontech Laboratories, Inc. (Mountain View, CA), Dynabeads Protein-G from Life Technologies (Grand Island, NY). Both goat anti-human IgG peroxidase antibody and human IgG were purchased from Sigma-Aldrich (St. Louis, MO).

### Isolation of ACPA and non-ACPA immune complexes

Since immunoglobulin IgG has two identical antigen binding sites, we reasoned that the ACPA occupied by a specific antigen at one site, potentially leaves the other unoccupied site available for immune isolation using the immobilized CCP3 peptide library included in the ELISA plates. Thus, we loaded synovial fluid samples onto commercial CCP3 ELISA plates and used this to isolate the ACPA immune complexes.

Synovial fluids were pre-treated with hyaluronidase (2 mg/ml) for 1 hour at room temperature to lower the viscosity then diluted 1:100 with 1% BSA-PBS for next step application. The ACPA levels were measured by anti-CCP3 ELISA, and the synovial fluids having high ACPA activity (>100 units) and zero activity were selected as ACPA positive and ACPA negative samples. The ACPA negative SF were further validated by mass spectrometry to have no immunoglobulin binding to CCP3. To isolate CCP3 reactive ACPA immune complexes, nineteen ACPA positive synovial fluids (100 μl) were added into each well of CCP3 ELISA plate and incubated for 45 min at room temperature. It was then washed 3 times with PBS plus 0.05% tween-20 and an additional 2 times washing with PBS. Trypsin (20 μl at 50 μg/ml) was added to the well and incubated at 37°C for 2 hours to allow protein digestion. The solutions in the wells were collected and lyophilized in 50% acetonitrile.

In recognition of the fact that the isolated ACPA IC included both antigen and non-antigen proteins, we also isolated IC from ACPA negative SF samples using protein-G. To isolate non-ACPA immune complexes, immunoprecipitations were performed by mixing ACPA negative synovial fluids (100 μl) with protein-G coupled magnetic beads (5 μl) at 4°C for 2 hours. After washing five times with PBS, the bound proteins were digested with trypsin at 37°C for 2 hours. The digested proteins were collected and lyophilized in 50% acetonitrile. ACPA negative SF was also added to CCP3 coated plate, the proteins that bind to CCP3 were used as antibody independent backgrounds.

### Mass spectrometry analysis

The identities of trypsinized proteins were analyzed on a LC/MS/MS mass spectrometry, TripleTOF 5600+ System (SCIEX, Ontario). Nano-flow LC Ultra system (Eksigent, Dublin, CA) packed with 3μm Luna C18 (Phenomenex, Torrance, CA) was inline installed. Samples were injected via a 300μm×5mm PepMap100 trap-column (ThermoFisher). Data acquisition of mass spectrometer were performed following settings: 250 ms survey MS spectra (m/z 380–1500) followed by up to 12 MS/MS measurements on the most intense parent ions (200 counts/sec threshold, +2 - +5 charge state, m/z single charged 400–1500 mass range for MS/MS, 100 ms each, high sensitivity mode).

Raw spectra files were converted into Mascot Generic File format (MGF) for peptide/protein identification by X!Tandem search algorithm (http://hs2.proteome.ca/tandem/thegpm_tandem_a.html). The X!Tandem search parameters were set at 20 ppm and 50 ppm mass tolerance for parent and fragment ions respectively, and two miss cleavage sites allowed [22]. The criterion of candidate molecule cut-off was: at least two matched peptides with Log(e) <-3, less than a thousandth probability of mismatching in database retrieval.

### Peptide Design

The citrullination of arginine makes the residue heavier by 0.984 Dalton and become resistant to trypsin digestion [23, 24]. Thus, we considered cleavable arginine to be un-citrullinated. We designed eight peptides containing arginine which cover all possible citrullination sites, at R3 (Cit3), R17 (Cit17), R19 (Cit19), R39 (Cit39), R40 (Cit40), and R95 (Cit95). The peptides were named according to the citrulline locations: Cit3, Cit17, Cit19, Cit39, Cit40, Cit39-40, Cit95, and no citrulline control peptide Pc corresponding to Cit39-Cit40. Each peptide consists of 13–15 amino acids with citrulline in the middle. Cit3: Met-Ser-Gly-Cit-Gly-Lys-Gly-Gly-Lys-Gly-Leu-Gly-Lys; Cit17: Gly-Lys-Gly-Gly-Ala-Lys-Cit-His-Arg-Lys-Val-Leu-Arg; Cit19: Gly-Gly-Ala-Lys-Arg-His-Cit-Lys-Val-Leu-Arg-Asp-Asn; Cit39: Ala-Ile-Arg-Arg-Leu-Ala-Cit-Arg-Gly-Gly-Val-Lys-Arg-Ile-Ser; Cit40: Ala-Ile-Arg-Arg-Leu-Ala-Arg-Cit-Gly-Gly-Val-Lys-Arg-Ile-Ser; Cit39-40: Ala-Ile-Arg-Arg-Leu-Ala-Cit-Cit-Gly-Gly-Val-Lys-Arg-Ile-Ser; Cit95: Ala-Leu-Lys-Arg-Gln-Gly-Cit-Thr-Leu-Tyr-Gly-Phe-Gly; Pc: Ala-Ile-Arg-Arg-Leu-Ala-Arg-Arg-Gly-Gly-Val-Lys-Arg-Ile-Ser.

All peptides were ordered from Biomatik Corporation (Cambridge, Ontario) with purity over 90%, the supplier performed mass spectrometry quality validation.

### ELISA

Anti-CCP3 assay was performed according to protocol with CCP3 kits. To test the antigenicities of citrullinated peptides by ELISA, each peptide was coated to an ELISA plate following the protocol of Takara peptide coating kit (MK100). Briefly, diluted peptide 50 μl (50 μg/ml) was added into 96 well plate plus 10 μl EDC (1-Ethyl-3-[3-dimethylaminopropyl] carbodiimide hydrochloride) provided by coating kit. After coating for 2 hours, the plate was blocked with BSA (2%) for 2 hours. Synovial fluid or serum diluted at 1:100 with 1%BSA-PBS was added and incubated for 45 minutes at room temperature. The plates were washed 3 times with PBS containing 0.05% tween 20. Goat anti-human IgG peroxidase antibody was added for 30 minutes. After washing five times, substrate 3,3,5,5′-Tetramethylbenzidine (TMB) was added to develop reactions, then the data were collected on a plate reader set at OD450 nm. Serially diluted human purified IgG was used to generate the standard curve for each ELISA assay. One unit of ACPA was defined as the signal level equal to 0.5 ng/well human IgG coating. The unit number in per milliliter sample was calculated and presented.

### Competitive Inhibition Assay

We tested the synovial fluid and serum samples at multiple dilutions and chose the most suitable dilution relative to the peptide concentration. The synovial fluid and serum were diluted at 1:800 with 1% BSA-PBS then equally divided into four tubes. Equal volume of PBS, control peptide (Pc), Cit19, or Cit39-40 was added respectively to each aliquoted sample at concentration (50mM). After incubation at room temperature for 20 minutes, the solutions were transferred to CCP3 ELISA plates to complete the procedure of ACPA tests. The ACPA activity in PBS treated sample was set at 100% (no competitive inhibition). The activities of peptide blocked sample was compared with PBS one to get the number of percentage inhibition.

## Results

### Characterization of *in vivo* citrullinated proteins binding to ACPA

Synovial and serum samples were categorized into ACPA positive and ACPA negative groups based on the anti-CCP3 ELISA assay. As described in the *Methods*, the ACPA IC isolated using the CCP3 ELISA were subjected to mass spectrometry to determine the spectrum of associated proteins, a proportion of which represented autoantigens that were bound specifically to the ACPA (S2 Table). In analyzing the IC that were isolated from ACPA negative SF samples using either CCP3 ELISA or protein-G, the proteins identified by mass spectrometry were considered to be non-antigenic (S3 Table and S4 Table, respectively). These two complementary approaches were used to eliminate protein candidates that did not bind the ACPA in an antigen specific manner, and a final list of candidate ACPA specific autoantigens is shown in Table 1. We reasoned that this subtractive approach, although conservative, was the most stringent for identifying *in vivo* antigens directly and specifically binding to the ACPA. Among nine proteins found in ACPA IC and deemed to be specific, histones were prominent, with H4 detectable in 17/19 (89%), H2B in 12/19 (63%), H3 in 12/19 (63%), and H2A in 11/19 (58%), and the other five proteins were identified only once in 19 (5%). These results suggest that histone H4, H2B, H3, and H2A, are likely the predominant endogenous citrullinated autoantigens directly binding to ACPA in RA synovial fluids. It should be added that citrullinated histones were not identified in association with serum derived ACPA IC. This distribution bias indicates that the synovial microenvironment is a site where ACPA are most likely to encounter citrullinated histones.

**Table 1:** Candidate autoantigens specifically bound to ACPA*.

Proteins	SF		Serum	
	ACPA IC (19)	Non-ACPA IC (9)	ACPA IC (8)	Non-ACPA IC (9)
HSP 27	1	0	0	0
Histone H2A	11	0	0	0
Histone H2B	12	0	0	0
Histone H3	12	0	0	0
Histone H4	17	0	0	0
Lactotransferrin	1	0	0	0
Myeloperoxidase	1	0	0	0
RAC2	1	0	0	0
Vimentin	1	0	0	0
Immunoglobulin heavy	19	9	8	9

* ACPA IC = CCP3 reactive immune complexes; non-ACPA IC = protein G reactive immune complexes from ACPA negative samples. The number in parentheses indicates the total samples tested, and the number in table body represents the frequency of identification in these samples.

### Antibodies to citrullinated histone 4 peptides in RA synovial fluid

**Fig 1:**
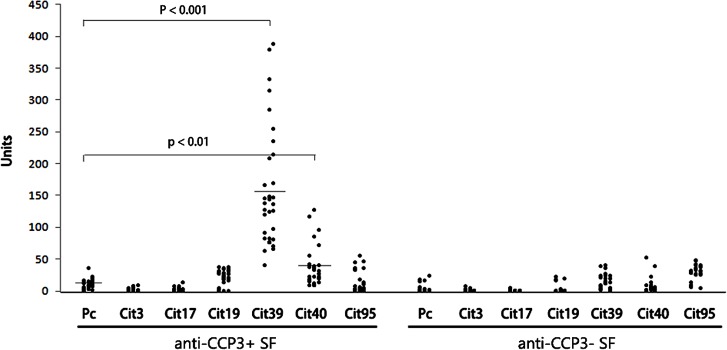
Reactivity to citrullinated histone H4 peptides in synovial fluids. Synthetic H4 peptides containing citrulline (Cit3, Cit17, Cit19, Cit39, Cit40, and Cit95) and control peptide (Pc) were coated to ELISA microplates, respectively, at 2.5 μg/well and blocked by BSA. Synovial fluids from anti-CCP3 positive RA (n = 34) and anti-CCP3 negative arthritis (n = 24) were tested for the levels of ACPA against each particular peptide. The mean value in each group is marked by a bar. The p-value was determined by comparing mean reactivity levels for each citrullinated peptide to the control peptide using t-test.

As histone H4 had the highest frequency of appearance in ACPA IC (89%), we selected histone 4 for detailed peptide characterization as described in the *Methods*. Synovial fluids from 34 ACPA positive RA and 24 ACPA negative arthritis patients were tested for reactivity against the anti-citrullinated H4 peptides immobilized on microplates. These results are shown in Fig 1. We set a cut off value for positivity as being above a level that excluded all but the top 2% of the reactivity to the control peptide (Pc) ELISA. Using this threshold, the positive reactions for ACPA positive SF samples to the H4 citrullinated peptides were: Cit3 (0%), Cit17 (0%), Cit19 (6%), Cit39 (100%), Cit40 (34%), and Cit95 (10%). Cit39 was the most frequently recognized, and was positive in 100% of SF samples, while Cit40 was recognized in 34% ACPA positive SF. None of the ACPA negative SF samples reacted with any of the H4 citrullinated peptides. These findings suggest that citrulline at position 39 of the H4 protein is important for recognition by ACPA in RA SF, while the citrulline at position 40 also contributes to the immune recognition.

### Anti-citrullinated H4 in the serum of RA patients and their first-degree relatives

**Fig 2:**
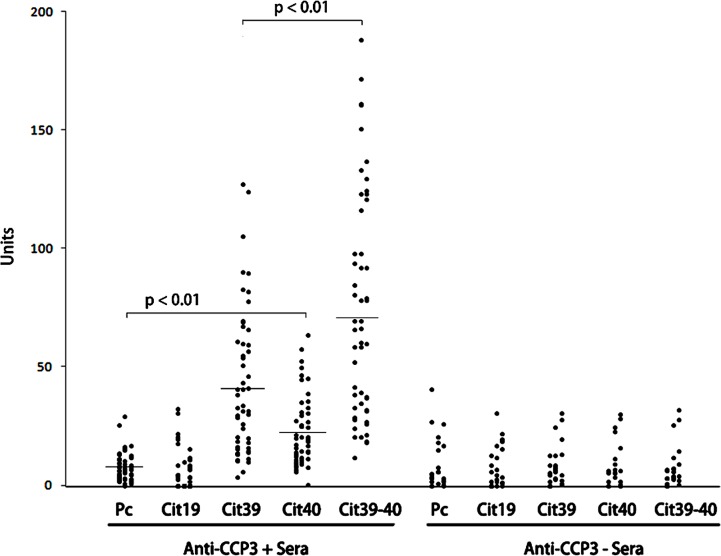
Reactivity to citrullinated histone H4 peptides in anti-CCP3 positive and negative sera. Sera from anti-CCP3 positive RA (n = 54) and anti-CCP3 negative healthy controls (n = 24) were tested for reactivity toward a number of citrullinated peptides derived from histone H4 (Cit19, Cit39, Cit40, Cit39-40), and control peptide (Pc). The p-value was determined by comparing mean reactivity levels for each citrullinated peptide to the control peptide using t-test.

We then tested serum samples from ACPA positive RA (n = 54) and ACPA negative healthy controls (n = 24). Based on the synovial fluid reactivity, we reasoned that a H4 peptide with a combination of Cit39 and Cit40 is likely to be the most antigenic, and we synthesized a peptide containing citrullines at R39 and R40 (Cit39-40). The results of ELISA assays using this peptide are shown in Fig 2. Again, the cutoff level was determined using responses to the control peptide (Pc) as described above. The frequency of reactivity in ACPA positive sera to a spectrum of citrullinated residues in the H4 protein was: Cit19 (4%), Cit39 (63%), Cit40 (38%), and Cit39-40 (87%). Thus, as predicted, Cit39-40 was the most frequently recognized by ACPA in the sera of RA patients. ACPA negative control sera did not show any reaction to the peptides.

**Fig 3:**
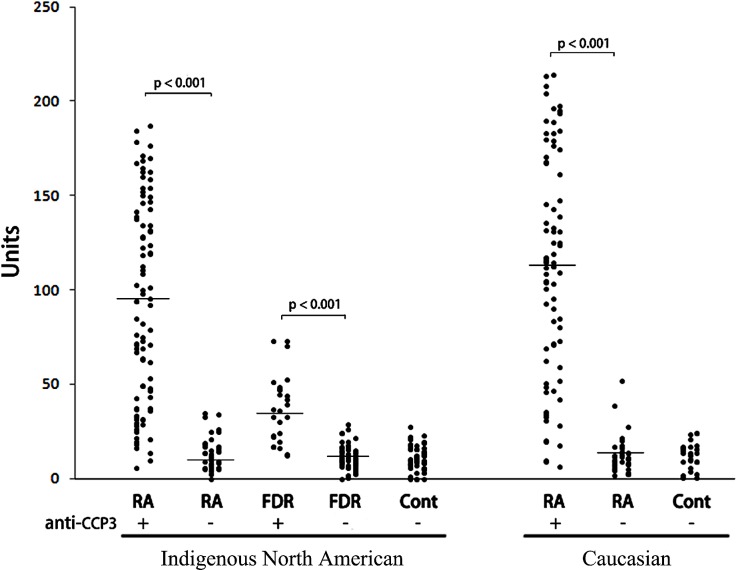
Reactivity to the histone H4 Cit39-40 peptide in a spectrum of ACPA positive and ACPA negative sera. Sera from anti-CCP3 positive Indigenous North American (INA) RA patients (n = 90), anti-CCP3 negative INA RA (n = 40), anti-CCP3 positive INA FDR (n = 26), anti-CCP3 negative INA FDR (n = 53), unrelated healthy INA controls (n = 54), anti-CCP3 positive Caucasian RA patients (n = 78), anti-CCP3 negative Caucasian RA patients (n = 38), and healthy Caucasian controls (n = 26), were tested for reactivity towards the citrullinated H4 Cit39-40 peptide. Mean levels of reactivity toward the Cit39-40 peptide were compared between anti-CCP3 positive and negative individuals in each group, and p-values represent significant differences as determined by t-test.

We next tested reactivity to the anti-Cit39-40 peptide in serum samples from Indigenous North American (INA) RA patients and their disease-free FDR who were enrolled in a longitidinal study of RA onset [19–21]. Both groups included ACPA positive and negative individuals as shown in S1 Table. In addition we tested samples from Caucasian ACPA positive and negative RA patients, and ACPA negative healthy controls from both INA and Caucasian populations. These data are shown in Fig 3. The results indicate that anti-Cit39-40 ACPA were detectable in the serum of 92% (83/90) of ACPA positive INA RA, 15% (6/40) of ACPA negative INA RA, 77% (20/26) of ACPA positive INA FDR, 7% (4/53) of ACPA negative INA FDR. On the other hand, anti-Cit39-40 ACPA were positive in the serum of 95% (74/78) of ACPA positive Caucasian RA patients, and 13% (5/38) of ACPA negative Caucasian RA patients. There were no obvious differences between INA and Caucasian RA patient populations, but the anti-cit39-40 levels detected in the INA ACPA positive FDR were generally lower compared to both INA and Caucasian RA patients. The results indicate that ACPA reactivity to H4 cit39-40 is present prior to disease onset, albeit at lower levels than in individuals with established disease.

### Histone H4 Cit39-40 competitively inhibits ACPA binding to CCP3

**Fig 4:**
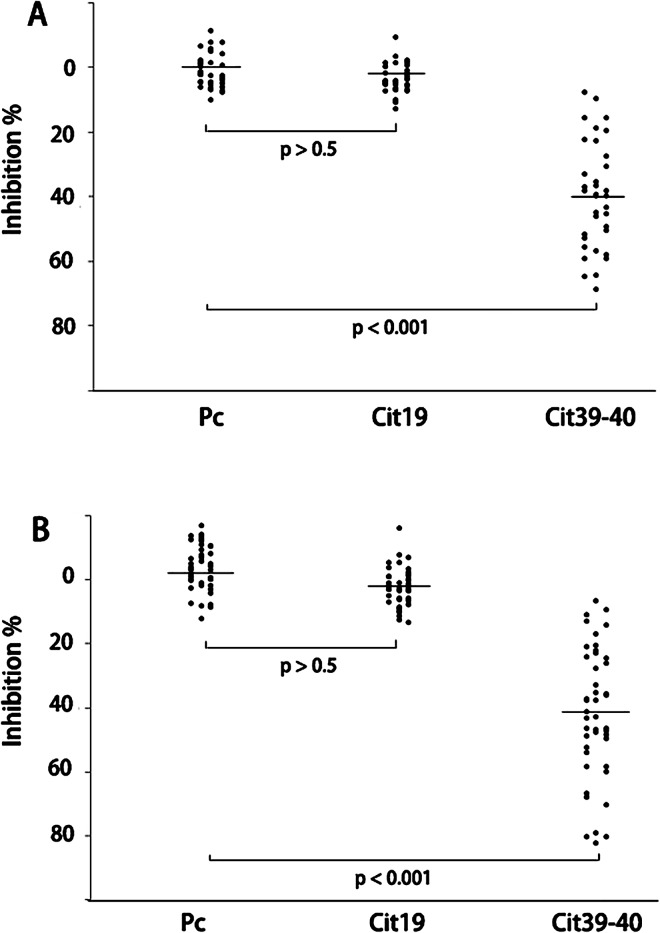
Histone H4 Cit39-40 peptide competitively inhibits anti-CCP3 reactivity in synovial fluid and serum. Equal volumes of anti-CCP3 positive synovial fluids (n = 35) and sera (n = 44) were premixed with 50mM of either a control peptide (Pc), peptide Cit19, peptide Cit39-40, or PBS for 20 minutes. These premixed samples were then tested for their anti-CCP3 reactivity. The ratio of each peptide treated sample to the PBS treated sample was used to calculate percentage inhibition. A) synovial fluid; B) serum. The p-value represents a significant difference between an H4 peptide and the Pc, using t-test.

We performed a competitive ELISA assay to determine the contribution of the anti-Cit39-40 antibodies to the total ACPA repertoire. In these experiments, ACPA positive SF (n = 35) and ACPA positive serum (n = 44) were tested as shown in Fig 4. The experimental procedure for these competitive assays are described in the Methods. Cit39-40 competitively inhibited ACPA reactions against CCP3 in all 35 synovial fluids ranged from 9–68% inhibition (average 40%). Peptide Cit19 showed minimal inhibitory effects ranging from 0 to 12.5%, with an average of 3%. Similar results were obtained for the serum samples with Cit39-40 competitively blocking ACPA reactions by 10–82% (average 41%). In contrast, Cit19 blocked ACPA responses by 0–13% (average 1.9%). These results demonstrated that anti-H4-cit39-40 antibodies comprise a substantial proportion of CCP3 reactive ACPA.

## Discussion

In this study, we used proteomic techniques in an attempt to identify *in vivo* citrullinated autoantigens that were binding directly and specifically to ACPA in RA synovial fluids, and in serum. The data we present highlight the predominance of histones, particularly histone H4, as endogenous autoantigens for ACPA in RA.

Our approach utilized an ACPA immune complex isolation strategy that was based on the assumption that these autoantibodies, in addition to binding a specific endogenous autoantigen on one arm, could also specifically bind a surrogate antigen included in the immobilized CCP3 array through the unoccupied antigen binding arm. The *in vivo* frequency of such mono-occupied antibodies with an available free arm is not known, and likely depends on the relative ratio of antibody to its cognate antigen. This ACPA isolation process is also inherently skewed towards the spectrum of immobilized citrullinated peptides which are included in the CCP3 plates. Although the composition of the CCP3 peptide array is proprietary, it is now widely accepted that this generation of CCP assay affords the highest level of clinical sensitivity and specificity to date [25, 26]. To ensure that the spectrum of autoantigens identified using the CCP3 isolation technique is broadly representative of the ACPA repertoire, we compared it to the proteins identified in protein-G captured total immune complexes. This further confirmed the predominance of histones in the ACPA positive samples (S5 Table).

A further assumption Inherent in our ACPA immune complex isolation and characterization strategy is the fact that there is likely a spectrum of proteins that are non-specifically bound to immunoglobulin *in vivo*, and that these proteins will be detected by mass spectrometry along with proteins that are specifically bound to the antigen binding arms. Thus, we used a stringent and conservative protocol of controls for eliminating the non-specifically bound proteins from further consideration. Although it can be argued that the combination of the CCP3 ACPA isolation procedure and the elimination of proteins deemed to be non-specifically bound has excluded important endogenous autoantigens from our analysis, we propose that this approach has afforded the highest level of specificity for *in vivo* autoantigen detection and characterization in our studies.

Previous studies have demonstrated the reactivity of RA sera with citrullinated histones exposed during the process of NETosis [27–31]. During this process, in which neutrophil DNA and nucleoproteins as released into the extracellular space and participate in host defense, histones are hypercitrullinated by PAD, and thus can become potential targets for ACPA. In experiments using purified PAD and recombinant Histone 4, Pratesi et al demonstrated that a histone 4 peptide containing 5 citrullines in sequence 31–50 was recognized by two thirds of the RA sera tested [30]. The data presented in our study of *in vivo* ACPA autoantigens are entirely consistent with these findings. Moreover, we further characterized Cit39 and Cit40 as being critical for the recognition of H4 by both synovial fluid and serum ACPA.

In addition to histones, our study did identify a number of other candidate proteins that are potential autoantigens. For example, fibrinogen (subunits α, β, γ) was identified in 16% of ACPA positive synovial immune complexes, but also in 44% of ACPA negative immune complexes isolated using protein-G (data not shown), suggesting that this protein may associate with ACPA both specifically as a citrullinated autoantigen, and as a non-antigen bound protein. There is now considerable evidence incriminating fibrinogen as a specific target for ACPA in RA, and it is widely detectable in the synovial microenvironment [15, 32, 33]. Other well characterized candidate autoantigens include vimentin, α-enolase, and collagen type II [5, 6, 10, 15, 32, 34]. Surprisingly, we identified vimentin in only 1/19 ACPA positive immune complexes from RA synovial fluids, and we did not identify α-enolase or collagen type II in any of the synovial immune complexes, whether they were isolated using protein-G or CCP3. The reasons for this discrepancy are unclear, but may point to important differences between ACPA autoantigens defined *in vitro* using purified antigens, and those detected *in vivo* in association with ACPA immune complexes. The potential for cross-reactivity in the *in vitro* assays remains an important cofounder, as exemplified by the broad reactivity to the surrogate cyclical antigens included on the CCP plates.

Despite considerable investigative effort, it remains unclear at what point histones, or any other endogenous proteins, become targeted as an autoantigen. It is now well recognized that ACPA are detectable in the serum of individuals who are destined to develop RA, months to years before synovitis is clinically detectable [13, 35]. Furthermore, there appears to be expansion of the spectrum of citrullinated proteins/peptides recognized by the ACPA as RA onset approaches, in a process termed epitope spreading [36, 37]. In the studies available to date, histone associated epitopes have been identified as candidate autoantigens [37, 38]. In the current study, we tested the sera of unaffected FDR of INA RA patients. These individuals are part of a longitudinal study of RA risk in INA [18, 21]. The data indicate that 77% (20/26) ACPA positive FDR (as determined by CCP3 positivity) recognize the Cit39-40 H4 peptide, albeit in lower titers than in RA patients, in whom it was recognized by 92% (83/90) of CCP3 positive serum samples. This indicates that the Cit39-40 H4 peptide is widely recognized by ACPA prior to the onset of RA synovitis. This is consistent with the finding that ACPA targeting a citrullinated histone H4 peptide (CitH431-50) were detectable in a subset of pre-symptomatic Swedish individuals who subsequently developed RA [39]. We speculate that with the onset of synovitis and the influx of neutrophils into RA joints, it is then likely that NETosis within the synovial compartment amplifies this response. Our competition experiments suggest that it becomes a predominant part of the ACPA repertoire in RA patients. Of note, the similar frequency and level of response between Caucasian and INA RA patients for the Cit39-40 H4 peptide suggest that this phenomenon is not unique to the INA population, and is broadly applicable across multiple RA populations.

An impediment to the development of tolerance inducing strategies for RA autoimmunity directed against citrullinated antigens has been the inability to define epitopes that are targeted *in vivo* in a substantial number of RA patients. The data presented in this study suggest that an epitope containing Cit39 and Cit40 of the histone H4 protein may be an appropriate candidate for further study in this regard, and that tolerance inducing strategies towards this epitope may be relevant even in the pre-clinical stages of RA.

## Supporting Information

S1 Table Subjects information. (DOCX)

S2 Table Proteins in CCP3 reactive ACPA immune complexes from ACPA positive synovial fluids. (DOCX)

S3 Table Synovial fluid proteins non-specifically bound to CCP3. (DOCX)

S4 Table Proteins detected in association with protein-G isolated immune complexes from ACPA negative synovial fluids. (DOCX)

S5 Table Proteins detected in association with protein-G isolated immune complexes from ACPA positive synovial fluids. (DOCX)
