# Supplementary material for: Characterization of Autoantigens Targeted by Anti-Citrullinated Protein Antibodies In Vivo: Prominent Role for Epitopes Derived from Histone 4 Proteins
Source: PLoS One. 2016 Oct 27;11(10):e0165501. doi: 10.1371/journal.pone.0165501 (PMC5082836; doi:10.1371/journal.pone.0165501)
Supplement: S1 Table — (DOCX) [file pone.0165501.s001.docx]

**S1 Table. Subjects information**

|  | INA RA |  | INA FDR | INA Ctrl | Cauc RA | Cauc Ctrl |
| --- | --- | --- | --- | --- | --- | --- |
| Total Number | 130 |  | 79 | 54 | 116 | 26 |
| Age Years mean(SD) | 46 (13) |  | 38 (13) | 32(11) | 59 (13) | 40 (11) |
| Female (% ) | 84 |  | 71 | 50 | 80 | 62 |
| CCP3 positive | 90 |  | 26 | 0 | 78 | 0 |
| Ever smoker (%) | 77 |  | 88 | 67 | 57 | 73 |
| HLA-DRB1 SE | 22/25 |  | 46/57 | 36/53 | NA | NA |

INA RA, Indigenous North American RA patient. INA FDR, Indigenous North American patient’s first degree relative. INA Ctrl, Indigenous North American healthy people. Cauc RA, Caucasian RA patient. Cauc Ctrl, Caucasian healthy people.
