# Supplementary material for: Characterization of Autoantigens Targeted by Anti-Citrullinated Protein Antibodies In Vivo: Prominent Role for Epitopes Derived from Histone 4 Proteins
Source: PLoS One. 2016 Oct 27;11(10):e0165501. doi: 10.1371/journal.pone.0165501 (PMC5082836; doi:10.1371/journal.pone.0165501)
Supplement: S2 Table — (DOCX) [file pone.0165501.s002.docx]

**S2 Table. Proteins in CCP3 reactive ACPA immune complexes from ACPA positive synovial fluids**

| **Ensembl#** | **Proteins** | **MW** | **Log(e)** |
| --- | --- | --- | --- |
| ENSP00000349960 | actin, beta | 41.7 | -36.7 |
| ENSP00000468139 | apolipoprotein C-II | 20 | -71.2 |
| ENSP00000295897 | albumin | 69.3 | -137.3 |
| ENSP00000323929 | alpha-2-macroglobulin | 163.2 | -22.5 |
| ENSP00000364469 | apolipoprotein A-I | 30.8 | -93.7 |
| ENSP00000356969 | apolipoprotein A-II | 11.2 | -24.3 |
| ENSP00000233242 | apolipoprotein B | 515.2 | -10.4 |
| ENSP00000465356 | apolipoprotein C-I | 9.3 | -57.5 |
| ENSP00000227667 | apolipoprotein C-III | 10.8 | -33.2 |
| ENSP00000252486 | apolipoprotein E | 36.1 | -461.6 |
| ENSP00000381250 | apolipoprotein F | 35.4 | -15.3 |
| ENSP00000244513 | butyrophilin | 58.9 | -7.9 |
| ENSP00000399421 | CD36 | 53 | -11.3 |
| ENSP00000418773 | ceruloplasmin | 108.8 | -29.9 |
| ENSP00000315130 | clusterin | 52.5 | -69 |
| ENSP00000363773 | complement component 1, q subcomponent, A chain | 26 | -19.1 |
| ENSP00000423689 | complement component 1, q subcomponent, B chain | 26.4 | -58.6 |
| ENSP00000363771 | complement component 1, q subcomponent, C chain | 25.8 | -43.1 |
| ENSP00000438615 | complement component 1, r subcomponent | 80.1 | -73.4 |
| ENSP00000385035 | complement component 1, s subcomponent | 76.6 | -172.2 |
| ENSP00000245907 | complement component 3 | 187 | -301.4 |
| ENSP00000356037 | complement component 4 binding protein, alpha | 67 | -6.4 |
| ENSP00000396688 | complement component 4A | 192.7 | -269.2 |
| ENSP00000415941 | complement component 4B | 192.6 | -276.7 |
| ENSP00000386770 | dynein | 514.5 | -6.1 |
| ENSP00000362817 | fatty acid binding protein 3 | 14.8 | -18.3 |
| ENSP00000306361 | fibrinogen alpha | 94.9 | -95.6 |
| ENSP00000306099 | fibrinogen beta | 55.9 | -55.8 |
| ENSP00000384860 | fibrinogen gamma | 49.5 | -69.9 |
| ENSP00000394423 | fibronectin 1 | 246.5 | -192.8 |
| ENSP00000348170 | haptoglobin | 45.2 | -70.7 |
| ENSP00000457629 | haptoglobin | 38.4 | -15.5 |
| ENSP00000441828 | haptoglobin-related protein | 39 | -82.6 |
| ENSP00000248553 | heat shock 27kDa protein | 22.8 | -76.7 |
| ENSP00000251595 | hemoglobin, alpha 2 | 15.2 | -40.3 |
| ENSP00000333994 | hemoglobin, beta | 16 | -78.1 |
| ENSP00000332194 | histone H2A | 14 | -106.3 |
| ENSP00000358164 | histone H2B | 13.9 | -85 |
| ENSP00000355657 | histone H3 | 15.5 | -132.2 |
| ENSP00000462667 | histone H4 | 11.4 | -146.3 |
| ENSP00000374989 | immunoglobulin heavy constant alpha 1 | 37.6 | -153 |
| ENSP00000374981 | immunoglobulin heavy constant alpha 2 | 36.6 | -68.4 |
| ENSP00000479178 | immunoglobulin heavy constant gamma 1 | 51.1 | -175.9 |
| ENSP00000481691 | immunoglobulin heavy constant gamma 1 | 52.4 | -149.8 |
| ENSP00000483022 | immunoglobulin heavy constant gamma 1 | 50.8 | -145 |
| ENSP00000375001 | immunoglobulin heavy constant mu | 49.4 | -178.4 |
| ENSP00000481881 | immunoglobulin heavy constant mu | 65.7 | -191.5 |
| ENSP00000484861 | immunoglobulin heavy constant mu | 64.1 | -392.3 |
| ENSP00000474284 | immunoglobulin heavy variable 1 | 13 | -32.6 |
| ENSP00000375014 | immunoglobulin heavy variable 1-18 | 12.8 | -30.9 |
| ENSP00000474941 | immunoglobulin heavy variable 3 | 12.6 | -20 |
| ENSP00000474363 | immunoglobulin heavy variable 3 | 10.7 | -60.8 |
| ENSP00000375010 | immunoglobulin heavy variable 3-11 | 12.9 | -52.1 |
| ENSP00000375012 | immunoglobulin heavy variable 3-15 | 12.9 | -44.3 |
| ENSP00000375024 | immunoglobulin heavy variable 3-33 | 13.1 | -55.8 |
| ENSP00000399826 | immunoglobulin heavy variable 3-43 | 13.1 | -18.2 |
| ENSP00000375034 | immunoglobulin heavy variable 3-49 | 13 | -17.7 |
| ENSP00000375036 | immunoglobulin heavy variable 3-53 | 12.8 | -34.2 |
| ENSP00000480175 | immunoglobulin heavy variable 3-64 | 8.3 | -12.5 |
| ENSP00000375041 | immunoglobulin heavy variable 3-66 | 12.7 | -28.5 |
| ENSP00000480035 | immunoglobulin heavy variable 3-72 | 11.2 | -44.5 |
| ENSP00000394447 | immunoglobulin heavy variable 3-74 | 12.8 | -34.3 |
| ENSP00000375025 | immunoglobulin heavy variable 4-34 | 13.8 | -64.7 |
| ENSP00000375028 | immunoglobulin heavy variable 4-39 | 13.9 | -55.1 |
| ENSP00000375035 | immunoglobulin heavy variable 5-51 | 12.7 | -37.1 |
| ENSP00000254801 | immunoglobulin J polypeptide | 18.1 | -21.1 |
| ENSP00000483856 | immunoglobulin kappa constant | 25.1 | -162.8 |
| ENSP00000478196 | immunoglobulin kappa constant | 25.6 | -135.3 |
| ENSP00000481923 | immunoglobulin kappa constant | 25.6 | -126.6 |
| ENSP00000374777 | immunoglobulin kappa constant I | 11.7 | -30.9 |
| ENSP00000420436 | immunoglobulin kappa variable 1-5 | 12.8 | -31.4 |
| ENSP00000420361 | immunoglobulin kappa variable 1-6 | 12.7 | -47.4 |
| ENSP00000480537 | immunoglobulin kappa variable 1-8 | 25.6 | -142.6 |
| ENSP00000419598 | immunoglobulin kappa variable 1-9 | 12.7 | -25.8 |
| ENSP00000480959 | immunoglobulin kappa variable 1D-13 | 12.6 | -27.5 |
| ENSP00000482678 | immunoglobulin kappa variable 1D-33 | 11.8 | -29.1 |
| ENSP00000419300 | immunoglobulin kappa variable 2-24 | 13.1 | -30.2 |
| ENSP00000419353 | immunoglobulin kappa variable 2-28 | 12.9 | -57.9 |
| ENSP00000482934 | immunoglobulin kappa variable 3-11 | 25.6 | -161.2 |
| ENSP00000418649 | immunoglobulin kappa variable 3-20 | 12.5 | -102.6 |
| ENSP00000374782 | immunoglobulin kappa variable 3-7 | 12.8 | -14.8 |
| ENSP00000374805 | immunoglobulin kappa variable 3D-20 | 12.5 | -58.3 |
| ENSP00000402914 | immunoglobulin kappa variable 3D-7 | 13.1 | -28.4 |
| ENSP00000374778 | immunoglobulin kappa variable 4-1 | 13.4 | -44.9 |
| ENSP00000374857 | immunoglobulin lambda constant 1 | 11.3 | -21.2 |
| ENSP00000374834 | immunoglobulin lambda variable 1-40 | 12.4 | -13.6 |
| ENSP00000374829 | immunoglobulin lambda variable 1-47 | 12.3 | -38.2 |
| ENSP00000374825 | immunoglobulin lambda variable 1-51 | 12.6 | -24.2 |
| ENSP00000374849 | immunoglobulin lambda variable 2-11 | 12.6 | -30.3 |
| ENSP00000374841 | immunoglobulin lambda variable 2-23 | 11.9 | -10.3 |
| ENSP00000374850 | immunoglobulin lambda variable 3-10 | 12.6 | -20.1 |
| ENSP00000374844 | immunoglobulin lambda variable 3-19 | 12 | -25.2 |
| ENSP00000374843 | immunoglobulin lambda variable 3-21 | 12.4 | -23.5 |
| ENSP00000374840 | immunoglobulin lambda variable 3-25 | 12 | -31.8 |
| ENSP00000374817 | immunoglobulin lambda variable 4-69 | 12.8 | -28.3 |
| ENSP00000374820 | immunoglobulin lambda variable 6-57 | 15.9 | -46.8 |
| ENSP00000482028 | immunoglobulin lambda-like polypeptide 5 | 24.8 | -51.1 |
| ENSP00000431254 | immunoglobulin lambda-like polypeptide 5 | 23 | -41.2 |
| ENSP00000252244 | keratin 1 | 66 | -193.1 |
| ENSP00000269576 | keratin 10 | 58.8 | -258.3 |
| ENSP00000167586 | keratin 14 | 51.5 | -34.7 |
| ENSP00000310861 | keratin 2 | 65.4 | -120.9 |
| ENSP00000252242 | keratin 5 | 62.3 | -54.5 |
| ENSP00000342710 | keratin 77 | 61.9 | -30.8 |
| ENSP00000246662 | keratin 9 | 62 | -101 |
| ENSP00000231751 | lactotransferrin | 78.1 | -7.6 |
| ENSP00000225275 | myeloperoxidase | 83.8 | -139.6 |
| ENSP00000216181 | myosin, heavy chain 9 | 226.4 | -10.4 |
| ENSP00000276914 | perilipin 2 | 48 | -8 |
| ENSP00000348888 | polymeric immunoglobulin receptor | 83.2 | -16.2 |
| ENSP00000249071 | Rac2 | 21.4 | -18.5 |
| ENSP00000271638 | S100 calcium binding protein A11 | 11.7 | -6.6 |
| ENSP00000357727 | S100 calcium binding protein A9 | 13.2 | -92.7 |
| ENSP00000390299 | serpin peptidase inhibitor, clade A | 46.7 | -26.7 |
| ENSP00000348918 | serum amyloid A1 | 13.5 | -51.1 |
| ENSP00000278222 | serum amyloid A4 | 14.7 | -52.1 |
| ENSP00000224237 | vimentin | 53.6 | -22.5 |
| ENSP00000362179 | zinc finger CCCH-type containing 12A | 65.7 | -6.5 |
