# Supplementary material for: Characterization of Autoantigens Targeted by Anti-Citrullinated Protein Antibodies In Vivo: Prominent Role for Epitopes Derived from Histone 4 Proteins
Source: PLoS One. 2016 Oct 27;11(10):e0165501. doi: 10.1371/journal.pone.0165501 (PMC5082836; doi:10.1371/journal.pone.0165501)
Supplement: S3 Table — (DOCX) [file pone.0165501.s003.docx]

**S3 Table. Synovial fluid proteins non-specifically bound to CCP3***

| **Ensembl#** | **Protein** | **MW** | **Log (e)** |
| --- | --- | --- | --- |
| ENSP00000392541 | albumin | 22.8 | -4.5 |
| ENSP00000295897 | albumin I | 69.3 | -17.7 |
| ENSP00000364469 | apolipoprotein A-I | 30.8 | -56.1 |
| ENSP00000252486 | apolipoprotein E | 36.1 | -23.1 |
| ENSP00000381250 | apolipoprotein F | 35.4 | -15.3 |
| ENSP00000380448 | apolipoprotein L | 43.9 | -8.1 |
| ENSP00000244513 | butyrophilin | 58.9 | -13.6 |
| ENSP00000386770 | dynein, heavy chain 1 | 514.5 | -5.1 |
| ENSP00000362817 | fatty acid binding protein 3 | 14.8 | -6.5 |
| ENSP00000251595 | hemoglobin, alpha 2 | 15.2 | -8.8 |
| ENSP00000357791 | hornerin | 282.2 | -10.9 |
| ENSP00000252244 | keratin 1 | 66 | -72.3 |
| ENSP00000269576 | keratin 10 | 58.8 | -101.3 |
| ENSP00000167586 | keratin 14 | 51.5 | -66 |
| ENSP00000310861 | keratin 2 | 65.4 | -144 |
| ENSP00000252242 | keratin 5 | 62.3 | -103.2 |
| ENSP00000342710 | keratin 77 | 61.9 | -26.5 |
| ENSP00000246662 | keratin 9 | 62 | -96.6 |
| ENSP00000276914 | perilipin 2 | 48 | -3.8 |
| ENSP00000417854 | protease, serine, 1 | 28.1 | -10 |
| ENSP00000357726 | S100 calcium binding protein A12 | 10.6 | -3.6 |
| ENSP00000348918 | serum amyloid A1 | 13.5 | -17.4 |
| ENSP00000278222 | serum amyloid A4 | 14.7 | -7.5 |
| ENSP00000362179 | zinc finger CCCH-type containing 12A | 65.7 | -4.4 |

*anti-CCP3 negative synovial fluid was added to the CCP3 plate and the proteins detected by mass spectrometry were considered to be background.
