# Supplementary material for: Characterization of Autoantigens Targeted by Anti-Citrullinated Protein Antibodies In Vivo: Prominent Role for Epitopes Derived from Histone 4 Proteins
Source: PLoS One. 2016 Oct 27;11(10):e0165501. doi: 10.1371/journal.pone.0165501 (PMC5082836; doi:10.1371/journal.pone.0165501)
Supplement: S4 Table — (DOCX) [file pone.0165501.s004.docx]

**S4 Table. Proteins detected in association with protein-G isolated immune complexes from ACPA negative synovial fluids**

| **Ensembl#** | **Protein** | **MW** | **Log(e)** |
| --- | --- | --- | --- |
| ENSP00000349960 | actin, beta | 41.7 | -27.7 |
| ENSP00000269143 | AFG3 ATPase family gene 3-like 2 | 88.5 | -3.4 |
| ENSP00000295897 | albumin | 69.3 | -315.3 |
| ENSP00000393887 | alpha-2-HS-glycoprotein | 39.3 | -3.3 |
| ENSP00000323929 | alpha-2-macroglobulin | 163.2 | -83.9 |
| ENSP00000355627 | angiotensinogen | 53.1 | -5.3 |
| ENSP00000364469 | apolipoprotein A-I | 30.8 | -157.4 |
| ENSP00000356969 | apolipoprotein A-II | 11.2 | -7.1 |
| ENSP00000350425 | apolipoprotein A-IV | 45.3 | -12.7 |
| ENSP00000233242 | apolipoprotein B | 515.2 | -18.2 |
| ENSP00000468139 | apolipoprotein C-II | 20 | -3.2 |
| ENSP00000227667 | apolipoprotein C-III | 10.8 | -67.9 |
| ENSP00000345179 | apolipoprotein D | 21.3 | -7.1 |
| ENSP00000386104 | carboxypeptidase E | 53.1 | -5.7 |
| ENSP00000360532 | cell division cycle 5-like | 92.2 | -4 |
| ENSP00000418773 | ceruloplasmin | 108.8 | -3.5 |
| ENSP00000315130 | clusterin | 52.5 | -10.7 |
| ENSP00000363773 | complement component 1, q subcomponent, A chain | 26 | -9.2 |
| ENSP00000423689 | complement component 1, q subcomponent, B chain | 26.4 | -38.9 |
| ENSP00000363771 | complement component 1, q subcomponent, C chain | 25.8 | -40.5 |
| ENSP00000444271 | complement component 1, r subcomponent | 41.3 | -5.7 |
| ENSP00000438615 | complement component 1, r subcomponent CUB domain | 80.1 | -57.8 |
| ENSP00000385035 | complement component 1, s subcomponent | 76.6 | -97.6 |
| ENSP00000245907 | complement component 3 | 187 | -137.1 |
| ENSP00000415941 | complement component 4B | 192.6 | -85.6 |
| ENSP00000263408 | complement component 9 | 63.1 | -20.5 |
| ENSP00000356399 | complement factor H | 139 | -3.3 |
| ENSP00000293371 | dermcidin | 11.3 | -7 |
| ENSP00000345739 | DNA (cytosine-5-)-methyltransferase 1 | 183 | -8.6 |
| ENSP00000386770 | dynein | 514.5 | -3.4 |
| ENSP00000356946 | Fc fragment of IgG | 32.7 | -5.2 |
| ENSP00000306361 | fibrinogen alpha | 94.9 | -9 |
| ENSP00000306099 | fibrinogen beta | 55.9 | -13.8 |
| ENSP00000384860 | fibrinogen gamma | 49.5 | -11.7 |
| ENSP00000394423 | fibronectin 1 | 246.5 | -81.7 |
| ENSP00000315106 | G protein-coupled receptor TM7SF1L2 | 47 | -3.1 |
| ENSP00000362929 | gelsolin | 80.6 | -31.5 |
| ENSP00000273951 | group-specific component | 52.9 | -12.1 |
| ENSP00000348170 | haptoglobin | 45.2 | -133 |
| ENSP00000441828 | haptoglobin-related protein | 39 | -75.6 |
| ENSP00000333994 | hemoglobin subunit beta | 16 | -3.1 |
| ENSP00000251595 | hemoglobin, alpha 2 | 15.2 | -19.3 |
| ENSP00000265983 | hemopexin | 51.6 | -24.1 |
| ENSP00000232003 | histidine-rich glycoprotein | 59.5 | -9.5 |
| ENSP00000374989 | immunoglobulin heavy constant alpha 1 | 37.6 | -4.2 |
| ENSP00000481691 | immunoglobulin heavy constant gamma 1 | 52.4 | -87.3 |
| ENSP00000374990 | immunoglobulin heavy constant gamma 1 | 43.9 | -301.3 |
| ENSP00000479178 | immunoglobulin heavy constant gamma 1 | 51.1 | -105.7 |
| ENSP00000481881 | immunoglobulin heavy constant mu | 65.7 | -52.8 |
| ENSP00000484861 | immunoglobulin heavy constant mu | 64.1 | -73.7 |
| ENSP00000375001 | immunoglobulin heavy constant mu | 49.4 | -64 |
| ENSP00000474284 | immunoglobulin heavy variable 1 | 13 | -12.1 |
| ENSP00000375014 | immunoglobulin heavy variable 1-18 | 12.8 | -20.3 |
| ENSP00000474363 | immunoglobulin heavy variable 3 | 10.7 | -49.3 |
| ENSP00000375010 | immunoglobulin heavy variable 3-11 | 12.9 | -57 |
| ENSP00000375012 | immunoglobulin heavy variable 3-15 | 12.9 | -7.3 |
| ENSP00000375024 | immunoglobulin heavy variable 3-33 | 13.1 | -40.8 |
| ENSP00000375034 | immunoglobulin heavy variable 3-49 | 13 | -31.6 |
| ENSP00000375036 | immunoglobulin heavy variable 3-53 | 12.8 | -19.8 |
| ENSP00000375041 | immunoglobulin heavy variable 3-66 | 12.7 | -18.8 |
| ENSP00000480035 | immunoglobulin heavy variable 3-72 | 11.2 | -27.3 |
| ENSP00000394447 | immunoglobulin heavy variable 3-74 | 12.8 | -10.2 |
| ENSP00000375021 | immunoglobulin heavy variable 4-28 | 13.1 | -5.1 |
| ENSP00000375035 | immunoglobulin heavy variable 5-51 | 12.7 | -38.6 |
| ENSP00000254801 | immunoglobulin J polypeptide | 18.1 | -7.5 |
| ENSP00000484499 | immunoglobulin kappa constant | 11 | -18.7 |
| ENSP00000478196 | immunoglobulin kappa constant | 25.6 | -102.4 |
| ENSP00000417427 | immunoglobulin kappa variable 1-37 | 12.7 | -5.7 |
| ENSP00000419058 | immunoglobulin kappa variable 1-39 | 12.7 | -44 |
| ENSP00000420436 | immunoglobulin kappa variable 1-5 | 12.8 | -15.8 |
| ENSP00000420361 | immunoglobulin kappa variable 1-6 | 12.7 | -56.8 |
| ENSP00000480537 | immunoglobulin kappa variable 1-8 | 25.6 | -126.4 |
| ENSP00000419598 | immunoglobulin kappa variable 1-9 | 12.7 | -41.9 |
| ENSP00000480959 | immunoglobulin kappa variable 1D-13 | 12.6 | -27.6 |
| ENSP00000482678 | immunoglobulin kappa variable 1D-33 | 11.8 | -43.6 |
| ENSP00000419300 | immunoglobulin kappa variable 2-24 | 13.1 | -5.8 |
| ENSP00000418138 | immunoglobulin kappa variable 2-30 | 13.2 | -18.9 |
| ENSP00000417637 | immunoglobulin kappa variable 2D-29 | 13.1 | -18.6 |
| ENSP00000482934 | immunoglobulin kappa variable 3-11 | 25.6 | -110.6 |
| ENSP00000418649 | immunoglobulin kappa variable 3-20 | 12.5 | -60.8 |
| ENSP00000374782 | immunoglobulin kappa variable 3-7 | 12.8 | -13.9 |
| ENSP00000374805 | immunoglobulin kappa variable 3D-20 | 12.5 | -21.4 |
| ENSP00000402914 | immunoglobulin kappa variable 3D-7 | 13.1 | -25.3 |
| ENSP00000374778 | immunoglobulin kappa variable 4-1 | 13.4 | -19.6 |
| ENSP00000374836 | immunoglobulin lambda variable 1-36 | 12.5 | -4.4 |
| ENSP00000374829 | immunoglobulin lambda variable 1-47 | 12.3 | -40.2 |
| ENSP00000374825 | immunoglobulin lambda variable 1-51 | 12.6 | -40.9 |
| ENSP00000374849 | immunoglobulin lambda variable 2-11 | 12.6 | -14.4 |
| ENSP00000374847 | immunoglobulin lambda variable 2-14 | 12.6 | -6.1 |
| ENSP00000374850 | immunoglobulin lambda variable 3-10 | 12.6 | -25.2 |
| ENSP00000374843 | immunoglobulin lambda variable 3-21 | 12.4 | -12.2 |
| ENSP00000374851 | immunoglobulin lambda variable 3-9 | 12.3 | -35.3 |
| ENSP00000374820 | immunoglobulin lambda variable 6-57 | 15.9 | -5.7 |
| ENSP00000374830 | immunoglobulin lambda variable 7-46 | 12.5 | -8.5 |
| ENSP00000374818 | immunoglobulin lambda variable 8-61 | 12.9 | -3.3 |
| ENSP00000414456 | immunoglobulin lambda variable 9-49 | 13 | -3.7 |
| ENSP00000431254 | immunoglobulin lambda-like polypeptide 5 | 23 | -14.9 |
| ENSP00000482028 | immunoglobulin lambda-like polypeptide 5 | 24.8 | -29.6 |
| ENSP00000252244 | keratin 1 | 66 | -139.8 |
| ENSP00000269576 | keratin 10 | 58.8 | -19.9 |
| ENSP00000167586 | keratin 14 | 51.5 | -6 |
| ENSP00000310861 | keratin 2 | 65.4 | -64.2 |
| ENSP00000252242 | keratin 5 | 62.3 | -73.8 |
| ENSP00000369317 | keratin 6A | 60 | -28 |
| ENSP00000252245 | keratin 75 | 59.5 | -8.6 |
| ENSP00000246662 | keratin 9 | 62 | -97.1 |
| ENSP00000299855 | matrix metallopeptidase 3 | 53.9 | -40.3 |
| ENSP00000259396 | orosomucoid 1 | 23.5 | -4.7 |
| ENSP00000394988 | patatin-like phospholipase domain containing 8 | 81.6 | -3 |
| ENSP00000252804 | peroxidasin homolog | 165.2 | -3.4 |
| ENSP00000308938 | plasminogen | 90.5 | -6.2 |
| ENSP00000427018 | regulating synaptic membrane exocytosis | 127.2 | -7.2 |
| ENSP00000365942 | RING finger protein 39 | 38.2 | -4.8 |
| ENSP00000376795 | serpin peptidase inhibitor, clade A | 47.6 | -7 |
| ENSP00000390299 | serpin peptidase inhibitor, clade A | 46.7 | -82.1 |
| ENSP00000215727 | serpin peptidase inhibitor, clade D | 57 | -4.3 |
| ENSP00000254722 | serpin peptidase inhibitor, clade F | 46.3 | -11.9 |
| ENSP00000278407 | serpin peptidase inhibitor, clade G | 55.1 | -3.1 |
| ENSP00000278222 | serum amyloid A4 | 14.7 | -6.8 |
| ENSP00000385834 | transferrin | 77 | -89.5 |
| ENSP00000237014 | transthyretin | 15.9 | -13.2 |
| ENSP00000348216 | tripartite motif-containing protein 4 | 57.4 | -3.2 |
| ENSP00000299427 | tripeptidyl peptidase I | 61.2 | -3.3 |
| ENSP00000418996 | Uncharacterized protein | 123.9 | -8.9 |
| ENSP00000410815 | Uncharacterized protein | 140.9 | -43.7 |
| ENSP00000339122 | VPS33B interacting protein | 57 | -3.6 |
| ENSP00000362179 | zinc finger CCCH-type containing 12A | 65.7 | -7.2 |
| ENSP00000424737 | zinc finger, GRF-type containing 1 | 236.5 | -5.5 |
