# Supplementary material for: Characterization of Autoantigens Targeted by Anti-Citrullinated Protein Antibodies In Vivo: Prominent Role for Epitopes Derived from Histone 4 Proteins
Source: PLoS One. 2016 Oct 27;11(10):e0165501. doi: 10.1371/journal.pone.0165501 (PMC5082836; doi:10.1371/journal.pone.0165501)
Supplement: S5 Table — (DOCX) [file pone.0165501.s005.docx]

**S5 Table. Proteins detected in association with protein-G isolated immune complexes from ACPA positive synovial fluids**

| **Ensembl #** | **Proteins** | **Mr (kDa)** | **log(e)** |
| --- | --- | --- | --- |
| ENSP00000349960 | actin, beta | 41.7 | -68.9 |
| ENSP00000295897 | albumin | 69.3 | -140.5 |
| ENSP00000323929 | alpha-2-macroglobulin | 163.2 | -25.2 |
| ENSP00000364469 | apolipoprotein A-I | 30.8 | -170.1 |
| ENSP00000356969 | apolipoprotein A-II | 11.2 | -18.9 |
| ENSP00000350425 | apolipoprotein A-IV | 45.3 | -81 |
| ENSP00000233242 | apolipoprotein B | 515.2 | -121.4 |
| ENSP00000465356 | apolipoprotein C-I | 9.3 | -9.7 |
| ENSP00000227667 | apolipoprotein C-III | 10.8 | -102 |
| ENSP00000345179 | apolipoprotein D | 21.3 | -10.2 |
| ENSP00000252486 | apolipoprotein E | 36.1 | -89.9 |
| ENSP00000418773 | ceruloplasmin | 108.8 | -35 |
| ENSP00000315130 | clusterin | 52.5 | -19.4 |
| ENSP00000423689 | complement component 1, q subcomponent | 26.4 | -45.5 |
| ENSP00000438615 | complement component 1, r subcomponent | 80.1 | -12.8 |
| ENSP00000385035 | complement component 1, s subcomponent | 76.6 | -48.7 |
| ENSP00000245907 | complement component 3 | 187 | -90.4 |
| ENSP00000396688 | complement component 4A | 192.7 | -37.9 |
| ENSP00000263408 | complement component 9 | 63.1 | -6.7 |
| ENSP00000257192 | desmoglein 1 | 113.7 | -16.8 |
| ENSP00000306361 | fibrinogen alpha | 94.9 | -197.2 |
| ENSP00000306099 | fibrinogen beta | 55.9 | -173.6 |
| ENSP00000384860 | fibrinogen gamma | 49.5 | -94.3 |
| ENSP00000392565 | fibronectin 1 | 26.5 | -35.5 |
| ENSP00000362929 | gelsolin | 80.6 | -24.1 |
| ENSP00000273951 | group-specific component | 52.9 | -10.7 |
| ENSP00000348170 | haptoglobin | 45.2 | -70.7 |
| ENSP00000265983 | hemopexin | 51.6 | -26.1 |
| ENSP00000332194 | Histone H2A | 14 | -50.2 |
| ENSP00000358164 | Histone H2B | 13.9 | -39.4 |
| ENSP00000355657 | Histone H3 | 15.5 | -10.5 |
| ENSP00000462667 | Histone H4 | 11.4 | -70.4 |
| ENSP00000277903 | hyaluronan binding protein 2 | 62.6 | -4.1 |
| ENSP00000374989 | immunoglobulin heavy constant alpha 1 | 37.6 | -88.3 |
| ENSP00000479178 | immunoglobulin heavy constant gamma | 51.1 | -165.8 |
| ENSP00000374987 | immunoglobulin heavy constant gamma 2 | 35.9 | -50.9 |
| ENSP00000481130 | immunoglobulin heavy constant gamma 3 | 56.9 | -101.4 |
| ENSP00000374985 | immunoglobulin heavy constant gamma 4 | 35.9 | -39.6 |
| ENSP00000484861 | immunoglobulin heavy constant mu | 64.1 | -240.1 |
| ENSP00000474363 | immunoglobulin heavy variable 3/OR16-9 | 10.7 | -56.7 |
| ENSP00000375012 | immunoglobulin heavy variable 3-15 | 12.9 | -31.5 |
| ENSP00000375024 | immunoglobulin heavy variable 3-33 | 13.1 | -42.3 |
| ENSP00000375034 | immunoglobulin heavy variable 3-49 | 13 | -36.1 |
| ENSP00000375036 | immunoglobulin heavy variable 3-53 | 12.8 | -15.8 |
| ENSP00000480035 | immunoglobulin heavy variable 3-72 | 11.2 | -53.9 |
| ENSP00000375035 | immunoglobulin heavy variable 5-51 | 12.7 | -30.4 |
| ENSP00000254801 | immunoglobulin J polypeptide | 18.1 | -17.9 |
| ENSP00000478196 | immunoglobulin kappa constant | 25.6 | -106.3 |
| ENSP00000374800 | Immunoglobulin Kappa light | 12.8 | -15.9 |
| ENSP00000420436 | immunoglobulin kappa variable 1-5 | 12.8 | -17.5 |
| ENSP00000480959 | immunoglobulin kappa variable 1D-13 | 12.6 | -20.1 |
| ENSP00000419300 | immunoglobulin kappa variable 2-24 | 13.1 | -11 |
| ENSP00000417637 | immunoglobulin kappa variable 2D-29 | 13.1 | -20.1 |
| ENSP00000482934 | immunoglobulin kappa variable 3-11 | 25.6 | -116.2 |
| ENSP00000418649 | immunoglobulin kappa variable 3-20 | 12.5 | -21.8 |
| ENSP00000374805 | immunoglobulin kappa variable 3D-20 | 12.5 | -40.6 |
| ENSP00000402914 | immunoglobulin kappa variable 3D-7 | 13.1 | -11.9 |
| ENSP00000374778 | immunoglobulin kappa variable 4-1 | 13.4 | -12.5 |
| ENSP00000374829 | immunoglobulin lambda variable 1-47 | 12.3 | -46.5 |
| ENSP00000374825 | immunoglobulin lambda variable 1-51 | 12.6 | -30.5 |
| ENSP00000374847 | immunoglobulin lambda variable 2-14 | 12.6 | -7.1 |
| ENSP00000374843 | immunoglobulin lambda variable 3-21 | 12.4 | -32.8 |
| ENSP00000431254 | immunoglobulin lambda-like polypeptide 5 | 23 | -44.9 |
| ENSP00000377507 | junction plakoglobin | 81.7 | -30.6 |
| ENSP00000252244 | keratin 1 | 66 | -114.5 |
| ENSP00000269576 | keratin 10 | 58.8 | -88.4 |
| ENSP00000167586 | keratin 14 | 51.5 | -34 |
| ENSP00000301653 | keratin 16 | 51.2 | -227.6 |
| ENSP00000310861 | keratin 2 | 65.4 | -136.7 |
| ENSP00000252242 | keratin 5 | 62.3 | -12.4 |
| ENSP00000369317 | keratin 6A | 60 | -266.9 |
| ENSP00000252250 | keratin 6C | 60 | -238.5 |
| ENSP00000329243 | keratin 7 | 51.4 | -26.6 |
| ENSP00000306261 | keratin 78 | 56.8 | -17.6 |
| ENSP00000467932 | keratin 9 | 39.5 | -134.3 |
| ENSP00000287611 | kininogen 1 | 47.9 | -3.9 |
| ENSP00000395337 | lactate dehydrogenase A | 36.7 | -4.1 |
| ENSP00000231751 | lactotransferrin | 78.1 | -13.3 |
| ENSP00000321334 | lipoprotein | 226.4 | -19.1 |
| ENSP00000298894 | Modulator of apoptosis 1 | 39.5 | -3.5 |
| ENSP00000225275 | myeloperoxidase | 83.8 | -5.2 |
| ENSP00000216181 | myosin, heavy chain 9 | 226.4 | -68.5 |
| ENSP00000237500 | myosin, light chain 12B | 19.8 | -8.7 |
| ENSP00000222381 | paraoxonase 1 | 39.7 | -15.3 |
| ENSP00000308938 | plasminogen | 90.5 | -11.4 |
| ENSP00000390299 | serpin peptidase inhibitor | 46.7 | -127.2 |
| ENSP00000348918 | serum amyloid A1 | 13.5 | -66.3 |
| ENSP00000256733 | serum amyloid A2 | 13.5 | -61.2 |
| ENSP00000278222 | serum amyloid A4 | 14.7 | -26.1 |
| ENSP00000427695 | transducer of ERBB2 | 38.1 | -3 |
| ENSP00000385834 | transferrin | 77 | -4.7 |
| ENSP00000237014 | transthyretin | 15.9 | -17 |
| ENSP00000339001 | tubulin, beta | 49.6 | -7.6 |
| ENSP00000224237 | vimentin | 53.6 | -8 |
| ENSP00000226218 | vitronectin | 54.3 | -25.5 |
| ENSP00000424737 | zinc finger, GRF-type containing 1 | 236.5 | -3.6 |
